# Hand Grip Strength Cut‐Off Points as a Discriminator of Sarcopenia and Sarcopenic Obesity: Results from the ELSA‐Brasil Cohort

**DOI:** 10.1002/jcsm.13723

**Published:** 2025-02-18

**Authors:** Clarice Alves Santos, Helena Fraga Maia, Francisco José Gondim Pitanga, Maria da Conceição Chagas de Almeida, Maria de Jesus Mendes da Fonseca, Estela Mota Leão de Aquino, Letícia de Oliveira Cardoso, Rosane Harter Griep, Sandhi Maria Barreto, Claudia Kimie Suemoto, Paulo Lotufo, Isabela Bensenor, Sheila Maria Alvim de Matos

**Affiliations:** ^1^ Department of Biological Sciences State University of the Southwest of Bahia Jequié Bahia Brazil; ^2^ Department of Life Sciences State University of Bahia Salvador Bahia Brazil; ^3^ Department of Physical Education Federal University of Bahia Salvador Bahia Brazil; ^4^ Oswaldo Cruz Foundation Instituto Gonçalo Moniz Salvador Bahia Brazil; ^5^ National School of Public Health Oswaldo Cruz Foundation Rio de Janeiro Brazil; ^6^ Institute for Collective Health Federal University of Bahia Salvador Bahia Brazil; ^7^ Laboratory of Health and Environment Education Oswaldo Cruz Institute Rio de Janeiro Brazil; ^8^ Faculty of Medicine & Clinical Hospital Federal University of Minas Gerais Belo Horizonte Minas Gerais Brazil; ^9^ Division of Geriatrics University of São Paulo, Faculdade de Medicina São Paulo Brazil; ^10^ Center for Clinical and Epidemiological Research University of São Paulo São Paulo Brazil

**Keywords:** diagnostic tests, muscular strength, musculoskeletal diseases, obesity, sarcopenia

## Abstract

**Background:**

Hand grip strength (HGS) may represent an epidemiologically relevant alternative as an initial screening tool for sarcopenia and sarcopenic obesity. However, no study evaluated the performance capacity of HGS compared to other biomarkers in discriminating these conditions in adults.

**Objective:**

The study aimed to evaluate the performance of HGS as discriminator of sarcopenia and sarcopenic obesity, compared to urinary biomarkers of creatinine and potassium in 24 h for Brazilian adults.

**Methods:**

Cross‐sectional study. Women (*n* = 5431) and men (*n* = 6351) aged 38–79 years who participated in the Brazilian Longitudinal Study of Adult Health (ELSA‐Brasil) at second follow‐up of the cohort (2012–2014). The area under the receiver operating characteristic curve (AUC) and the respective 95% confidence intervals were calculated for men and women in different age groups to assess the performance of HGS as a discriminator of sarcopenia and sarcopenic obesity, compared to the biomarkers of potassium and creatinine in urine in 24 h. The outcomes were classified based on the skeletal muscle mass index (BMI/height^2^) and fat percentage, estimated from the bioimpedance analysis data. Sensitivity, specificity and Brier score were calculated for each estimated HGS cut‐off point.

**Results:**

It can be observed that 18.20% (15.51% women; 21.34% men) of the population showed a decline in skeletal muscle mass (sarcopenia). Of this total, 11.61% (10.52% women; 12.89% men) presented the isolated outcome of sarcopenia and 6.59% (4.99% women; 8.45% men) of sarcopenic obesity. The HGS areas under the ROC Curve ranged from 0.54 (CI = 0.493–0.596) to 0.76 (CI = 0.650–0.878) according to sex and age group. HGS performance compared to biomarkers was significantly higher in virtually all strata and outcomes analysed. The cut‐off points that demonstrated greater accuracy and better performance in outcome discrimination were ≤ 42, ≤ 41, ≤ 38 and ≤ 36 kgf among males aged 38–44 years, 45–54 years, 55–64 years and 65–79 years, respectively. For women in the same age groups, HGS cut‐offs were ≤ 26, ≤ 23, ≤ 23 and ≤ 21 kgf, respectively.

**Conclusions:**

The results suggest that HGS is a good discriminator of sarcopenia and sarcopenic obesity, capable of achieving superior or equal performance to muscle mass biomarkers, especially in middle‐aged adults.

## 1 Introduction

In social contexts characterized by the rapid aging of the population and the obesity epidemic, evaluating body composition throughout the lifespan becomes a crucial health measure for monitoring mortality risk [1, 2, 3]. Additionally, it serves as a diagnostic tool for early intervention in diseases and conditions associated with skeletal muscle changes, such as sarcopenia [1, 4]. Defined by the involuntary and progressive decline of muscle mass, muscle strength and/or function [4, 5], sarcopenia may also be accompanied by an increase in fat mass, especially in the abdominal region, constituting a condition called sarcopenic obesity [6, 7].

Among the methods of evaluation of body components widely used as a screening tool for the diagnosis of sarcopenia, there are indirect methods such as computed tomography, magnetic resonance imaging, dual‐energy x‐ray absorptiometry (DEXA) and creatinine and potassium biomarkers, and doubly indirect methods, such as electrical bioimpedance (BIA) and anthropometry [8, 9, 10].

The HGS, as a measure of muscle quality and marker of general health status [1, 2, 11, 12], has also been pointed as a valid and reliable measure to discriminate changes in skeletal muscle mass [9, 13, 14]. The indication of its use as an initial screening tool for sarcopenia has been supported by the European Working Group on Sarcopenia in Older People (EWGSOP) [5], who consensually recommend the use of strength and skeletal muscle mass criteria together for the diagnosis of this condition in populations. Additionally, both highlight the need for standardization of methods and classification criteria, as well as the adjustment of specific cut‐off points in the studied populations, with the aim of promoting a universal definition and providing more accurate and comparable estimates with studies conducted in other sociocultural contexts.

However, in Latin American countries, there is a scarcity of normative data for the criteria of strength and muscle mass suggested by the EWGSOP, with reference to the adult population of these countries. In this sense, establishing normative values for normal decline muscle mass and sarcopenia, as well as estimating specific HGS cut‐off points as a discriminator of these conditions in Brazilian populations, may indicate a simple and relevant alternative as an initial screening tool for changes in skeletal muscle mass in this population, in addition to contributing to a universal definition and providing more accurate estimates comparable with studies conducted in other sociocultural contexts.

In primary health care, the incorporation of this indicator into care protocols may favour the anticipation of health promotion strategies in a more adequate way to the population, establishing evidence for the management of health priorities, given the high demand of older and middle‐aged adult users who may benefit from early diagnosis and treatment of sarcopenia.

The aim of this study was to evaluate the performance of HGS as a discriminator of sarcopenia and sarcopenic obesity classified based on the skeletal muscle mass index (SMMI), compared to the performance of urine creatinine and potassium biomarkers in 24 h biomarkers among participants of the Brazilian Longitudinal Study of Adult Health (ELSA‐Brasil).

## 2 Materials and Methods

### 2.1 Study Design and Participants

The ELSA‐Brasil is a cohort of men and women aged 35–74 years old in as six Brazilian cities (Belo Horizonte, Salvador, Rio de Janeiro, São Paulo, Vitória). This one study of a follow‐up performed of adults in Latin America, with the main purpose of investigating chronic diseases, such as diabetes and cardiovascular diseases, and their risk factors, according to the previously described methodology [15]. It should be noted that ELSA‐Brasil had its research protocol submitted and approved by the National Commission for Research Ethics (CONEP n° 13 065) and by the ethics committees of each institution, meeting all the ethical requirements necessary for research involving human beings.

The present study is based on data collected in the second follow‐up of the ELSA‐Brasil (2012–2014) which examined 14 014 people. Participants without information of the bioelectrical impedance analysis (BIA) (*n* = 939), anthropometric measures of weight and height and HGS (*n* = 569) were excluded. Subjects with limb amputations or those with metal prostheses (*n* = 585) were excluded from the analysis, which could compromise the verification of measurements and the accurate estimation of body mass through BIA and HGS. Individuals identified in the database with values outside the measurement range allowed by the device (HGS < 0 and > 90 kgf) (*n* = 1) were excluded. Participants who had undergone bariatric surgery (*n* = 138) were also excluded from the analysis due to the marked decline in strength parameters and body composition that occurred after the surgery. After exclusions, the final sample consisted of 11 782 (84.1%) of participants from the second follow‐up of the study.

An additional exclusion criterion was applied for the 1181 participants aged less than or equal to 40 years at the baseline and less than 45 years at the second follow‐up. A total of 474 participants who self‐reported a previous diagnosis of diseases such as diabetes, cancer, acute myocardial infarction, angina, heart failure, cardiac surgery, renal disease, asthma, emphysema, bronchitis, chronic obstructive pulmonary disease, cirrhosis and arthritis study. Participants who reported hospitalization in the last 12 months were also excluded from the group. These criteria were used to estimate reference values for normal muscle mass classification and sarcopenia based on the healthy young adult population of the study. The estimated values were later applied to construct the outcomes analysed in the total study population.

### 2.2 Data Collection

ELSA‐Brasil data were collected in the research centers of each institution, with standardization of the scientific criteria [15, 16]. Sociodemographic information obtained through face‐to‐face interviews using a multidimensional questionnaire, standardized for the study (16); anthropometric measures (body mass and stature) and electrical bioimpedance analysis (skeletal muscle mass and percentage of body fat), measured according to the standards and techniques recommended by Lohman [17].

Body mass (kg) was determined using an electronic balance of the brand Toledo, with a maximum capacity of 200 kg and an accuracy of 0.1 kg. The stature in (cm), later converted to (m), was measured using a fixed stadiometer of the brand SECA (Model SE‐216) and scale of 0.1 cm.

The skeletal muscle mass (SMM) and the body fat percentage (BF) were measured with the BIA using InBody 230 (Biospace Co., Ltd, Seoul, Korea), an octopolar (eight electrodes) analyser of body composition and operating frequency of 20 and 100 KHz at the passage of an electric current not perceptible by the subject examined. This test demonstrates a good correlation with DEXA (*r* = 0.94–0.99) [18] and was included into the data collection protocol of the second follow‐up of the ELSA‐Brasil cohort.

For the BIA examination, participants were instructed to empty the bladder and remove all metallic props. The test was performed with the overnight fasting patient from 12 to 15 h and in an air‐conditioned environment.

The HGS was included in the second follow‐up data collection protocol and measured using the Jamar hydraulic hand dynamometer, with a maximum capacity of 90 kgf and 5 adjustment positions for the hand size (Sammons Preston Rolyan, Bolingbrook, IL). The examination was carried out with three measurements in each hand, starting at the dominant hand and alternating then to the non‐dominant hand, with the participant comfortably seated in a chair with a back and aid support for the forearm, with the shoulder slightly adducted, elbow bent at 90° and thumb facing up. For standardization of HGS (hand grip) measurements, a single adjustment was adopted at the top (position 3) and at the bottom (position 2) of the instrument handle. During the execution of the test, the participant was verbally encouraged to print as much strength as possible on each hand. For the analysis of the data, it was considered the best strength performance obtained between the three measures performed in each member [19].

Biochemical markers indicated of alteration in skeletal muscle mass, such as creatinine (mg/dL) and potassium (mEq/L) in 24‐h urine samples, were obtained from participants after a 12‐h overnight fasting [20].

### 2.3 Predictor Variables

The HGS, the main predictor variable, and levels of potassium and creatinine were tested as discriminant of the outcomes sarcopenia and sarcopenic obesity, classified based on SMMI. These biomarkers were used because they are related to muscle metabolism and are considered indirect markers of muscle tissue quantity and quality [8, 10].

### 2.4 Outcome Variables

Anthropometric indicators of body mass and height such as SMM and BF measures were used to construct the outcomes of interest in the present study. These measures were necessary to calculate the SMMI adjusted by the square of the height in meters (SMM/height^2^), proposed by Janssen et al. in a study with data from the Third National Health and Nutrition Examination Survey (NHANES III) as criterion for classification of normal muscle mass and sarcopenia [21].

Sarcopenia was classified based on the mean of the SMMI sex‐specific distribution in healthy young adults aged less than or equal to 40 years old at the baseline and less than 45 years at the second follow‐up of ELSA‐Brasil. The choice of this age group as a reference was due to the evidence that the decline in skeletal muscle mass and muscle strength throughout life is detectable mainly after the age of 45 and is progressively increasing in subsequent years [22].

Participants who obtained SMMI values < 1 standard deviations from the mean value of the reference group, regardless of body fat percentage were classified as sarcopenic. This classification criterion of the SMMI is similar to that used by the World Health Organization to classify osteoporosis and osteopenia [23].

An isolated condition of sarcopenia was considered to be present among individuals with SMMI < 1 standard deviation of the mean value of the reference group of young adult and BF lower than the median specific sex in the population of the present study. The isolated occurrence of sarcopenic obesity was considered among those whose sharp decline in SMMI was also accompanied by a BF higher than the value of the median specific sex obtained in the total study population [24].

### 2.5 Data Analysis

All analyses were performed using the STATA software, Version 14.0. The analyses were conducted and presented separately by sex, considering, a priori, the differences in the body structures of men and women. The Shapiro–Wilk test was employed to assess the normality of the variables. Pearson's chi‐square test was used for the comparison of frequencies, and the unpaired Student's *t*‐test was applied to compare means. A one‐way analysis of variance (ANOVA) was performed, followed by a Bonferroni post‐hoc test to estimate differences in the means of the assessed indicators across the age categories (≤ 44 years, 45–55 years, 55–64 years and ≥ 65 years) in both men and women. The statistical significance level was set at 5%.

The areas under the receiver operating characteristic (ROC) curve and their respective confidence intervals (95% CI) were calculated and tested to assess the discriminatory capacity of the diagnostic test models [25] between the predictive variable (HGS) and the outcome of sarcopenia and sarcopenic obesity, comparing them with of potassium and creatinine in the urine in 24‐h biomarkers.

Sequentially, three strategies were used to estimate the ‘optimal’ cut‐off points for HGS. The first, the Youden index (J) is an indication of the best overall test performance, defined as max (sensitivity + specificity − 1). This index maximizes the general rate of correct classification and makes it possible to identify the farthest point of the area of uncertainty/chance or not significance. The second strategy used was based on the closest cut‐off point of the ideal, represented by the smallest distance squared (d^2^) between the ROC curve and the upper left corner of the graph, which shows sensitivity and perfect specificity [26]. The third strategy, based on the cut‐off point whose area under the ROC curve, demonstrates a better balance between sensitivity and specificity (e). In addition, sensitivity and specificity values were estimated for all HGS cut‐offs and Brier scores [27] to evaluate the accuracy of the prediction models suggested by the strategies employed.

Logistic regression analysis was used to verify the associations between HGS cut‐off points and sarcopenia/sarcopenic obesity, with the crude odds ratio (OR) being calculated and the corresponding 95% CI.

## 3 Results

5431 men and 6351 women of similar mean age between the sexes (55.6 years ± 9.0) were included in the present study. This population was distributed in four age groups from 38 to 44 years old (10.54%); 45 to 54 years (38.14%); and 55 to 64 years (34.05%) and 65 to 79 years (17.26%), the youngest group being considered as a reference for the classification of normal skeletal muscle mass, sarcopenia and sarcopenic obesity.

The reference values for muscle mass decline were less than one standard deviation of the mean number of SMMI in young adults, with values lower than 10.03 kg m^2^ for men and 8.00 kg m^2^ for women considered in the classification of sarcopenia. SMMI values (kg/m^2^) above the reference points mentioned and a body fat percentage greater than the median specific sex in the total study population (BF ≤ 29% for men and ≤ 38% for women) were used to classify sarcopenic obesity and are shown in the Table 1.

**TABLE 1:** Characteristics of the size and body composition of the population of younger and healthy adult men and women, considered as a reference for classification of normal muscle mass, sarcopenia and sarcopenic obesity. ELSA‐Brasil, 2012–2014.

Variables	Younger, healthier adult population (reference)^a^	
	MEN (*n* = 361)	WOMEN (*n* = 346)
	Average (±SD)	Average (±SD)
Weight (kg)	82.34 (13.90)	69.90 (14.55)
Height (m)	1.74 (0.06)	1.61 (0.06)
Skeletal muscle mass (kg)	34.34 (4.68)	23.73 (3.51)
Appendicular fat‐free mass (kg)	25.78 (3.38)	17.75 (2.74)
Appendicular muscle mass index (kg)	8.40 (0.75)	6.78 (0.79)
Skeletal muscle mass index (kg)	11.20 (1.17)	9.08 (1.08)
Skeletal muscle mass index in percentage (%)	42.08 (3.86)	34.50 (3.81)
Body fat percentage (%)	25.57 (6.86)	36.74 (7.19)
Hand grip strength (kgf)	45.27 (10.08)	27.32 (7.33)
Potassium (mEq/L)	33.47 (18.33)	31.93 (16.31)
Creatinine (mg/dL)	122.99 (63.74)	93.77 (46.71)

Abbreviations: AFFM (kg) = appendicular fat free mass in kilogram, AMMI (kg) = appendicular muscle mass index in kg (AFFM/height^2^), BF (%) = body fat percentage, HGS = hand grip strength in kilogram/strength, SD = standard deviation, SMM (kg) = skeletal muscle mass in kilograms, SMMI (%) = skeletal muscle mass index in percentage (SMM/weight) × 100, SMMI (kg) = skeletal muscle mass index in kg (SMM/height^2^).

^a^ Reference group of younger, healthier adults aged ≤ 40 years at baseline and aged < 45 years in the second cohort data collection (2012–2014), without diagnoses of diseases and self‐reported illnesses and hospitalization in the last 12 months, which preceded the ELSA‐Brasil baseline data collection;

The SMMI calculated based on SMM were significantly higher in males, whereas BF was higher in females. It can be observed that 18.20% (15.51% women; 21.34% men) of the population showed a decline in skeletal muscle mass (sarcopenia). Of this total, 11.61% (10.52% women; 12.89% men) presented the isolated outcome of sarcopenia and 6.59% (4.99% women; 8.45% men) of sarcopenic obesity. The prevalence of the isolated or aggregated sarcopenia and sarcopenia obesity, classified on the basis of the SMMI, increased with age for both sexes, as shown in Table 2.

**TABLE 2:** Characteristics of body size and composition, muscular strength and prevalence of sarcopenia/sarcopenic obesity, according to the age range in men of women. ELSA‐Brasil, 2012–2014.

Variables	Men				Women			
	38–44 years *n* = 593	45–54 years *n* = 2097	55–64 years *n* = 1779	65–79 years *n* = 962	38–44 years *n* = 649	45–54 years *n* = 2397	55–64 years *n* = 2233	65–79 years *n* = 1072
Weight (kg)	83.03 (±14.84)	82.89 (±14.73)	80.52 (±14.13)	77.52 (±13.29)	70.47 (±14.75)	70.71 (±13.90)	69.65 (±13.38)	67.51 (±12.56)
Height (m)	174.92 (±6,80)	172.68 (±6,93)	171.14 (±6,99)	169.11 (±7,01)	161.51 (±6.31)	159.76 (±6.23)	157.57 (±6.25)	155.55 (±6.17)
SMM (kg)	34.55 (±5.01)	33.59 (±4.73)	31.96 (±4.58)	29.89 (±4.48)	23.81 (±3.52)	23.44 (±3.49)	22.51 (±3.32)	21.38 (±3.17)
AFFM (kg)	25.91 (±3.62)	25.14 (±3.56)	23.95 (±3.50)	22.42 (±3.53)	17.82 (±2.77)	17.43 (±2.74)	16.60 (±2.63)	15.52 (±2.59)
AMMI (kg)	8.43 (±0.79)	8.39 (±0.80)	8.14 (±0.80)	7.79 (±0.82)	6.80 (±7.93)	6.80 (±8.01)	6.63 (±7.86)	6.38 (±6.38)
SMMI (kg)	11.25 (±1.21)	11.23 (±1.16)	10.88 (±1.13)	10.41 (±1.10)	9.10 (±1.07)	9.16 (±1.07)	9.01 (±1.04)	8.81 (±1.02)
SMMI (%)	41.99 (±3.90)	40.92 (±3.82)	40.05 (±3.62)	38.86 (±6.62)	34.33 (±3.86)	33.61 (±3.78)	32.74 (±3.60)	32.04 (±3.43)
BF (%)	25.73 (±6.90)	27.38 (±6.79)	28.44 (±6.41)	29.84 (±6.48)	37.07 (±7.27)	38.30 (±6.98)	39.52 (±6.73)	40.29 (±6.42)
HGS (kgf)	45.48 (±10.64)	43.68 (±9.56)	40.88 (±8.61)	36.84 (±9.11)	27.06 (±7.27)	26.13 (±6.44)	24.68 (±6.13)	22.84 (±5.66)
Potassium (mEq/L)	33.72 (±18.60)	33.09 (±17.90)	32.65 (±17.32)	36.98 (±18.28)	31.69 (±16.58)	29.41 (±15.38)	27.87 (±14.98)	28.57 (±13.94)
Creatinine (mg/dL)	122.24 (±62.00)	110.79 (±55.52)	96.76 (±49.46)	86.20 (±41.63)	93.51 (±48.89)	79.06 (±42.45)	64.55 (±36.48)	56.73 (±29.75)

	*n* (%)	*n* (%)	*n* (%)	*n* (%)	*n* (%)	*n* (%)	*n* (%)	*n* (%)
Sarcopenia^a^	74 (12.48)	221 (10.54)	257 (14.45)	148 (15.38)	73 (11.25)	210 (8.76)	227 (10.16)	158 (14.74)
Sarcopenic obesity^b^	18 (3.04)	77 (3.67)	155 (8.71)	209 (21.73)	17 (2.62)	93 (3.88)	125 (5.60)	82 (7.65)
Sarcopenia and sarcopenic obesity^c^	92 (15.51)	298 (14.21)	412 (23.16)	357 (37.11)	90 (13.87)	303 (12.64)	352 (15.76)	240 (22.39)

*Note:* Data presented as mean (± standard deviation), unless otherwise indicated. Pearson's chi‐square test was used to compare frequencies, and unpaired Student's *t*‐test was used to compare means (*p* < 0.001). One‐way ANOVA with Bonferroni post hoc test to estimate differences in the means of the indicators evaluated according to gender and age group and considered significant when *p* < 0.05.

Abbreviations: AFFM (kg) = appendicular fat free mass in kilogram, AMMI (kg) = appendicular muscle mass index in kg (AFFM/height^2^), BF (%) = fat percentage, HGS = hand grip strength in kilogram/strength, SMM (kg) = skeletal muscle mass in kilograms, SMMI (%) = muscle mass index in percentage (SMM/weight) × 100, SMMI (kg) = muscle mass index in kg (SMM/heigh^t2^).

^a^ Sarcopenia (isolated condition of SMMI < 1 standard deviation of the mean healthy young reference population).

^b^ Sarcopenic obesity (isolated condition of SMMI < 1 standard deviation of the mean young reference population and BF > median specific sex in the total study population).

^c^ Sarcopenia and sarcopenic obesity (combined outcome of sarcopenia + sarcopenic obesity).

The values of the area on the HGS curve for discrimination of the isolated and combined outcomes of sarcopenia and sarcopenia obesity ranged from 0.544 (CI = 0.493–0.596) to 0.764 (CI = 0.650–0.878) for men and women, the largest areas being verified for the isolated outcome of sarcopenic obesity and the lowest for the isolated sarcopenia condition. It can be observed a better performance of HGS in the discrimination of sarcopenia and sarcopenic obesity when compared to potassium and creatinine biomarkers in the urine in 24 h, with significantly larger areas in all strata of gender and age group. The best HGS performance was also observed for the isolated outcomes of sarcopenia and sarcopenic obesity, except in the 65–79 age group, whose performance was higher for the creatinine and potassium markers for men and for creatinine among women. These results are shown in Table 3.

**TABLE 3:** Areas under the ROC curve and respective confidence intervals (CI95%) of HGS and biomarkers of muscle mass as discriminator of sarcopenia and sarcopenic obesity, according to the age range in men and women. ELSA‐Brasil, 2012–2014.

Muscle mass marker	Sarcopenia (isolated)				Sarcopenic obesity (isolated)				Sarcopenia and sarcopenic obesity			
	38–44 years	45–54 years	55–64 years	65–79 years	38–44 years	45–54 years	55‐64 years	65–79 years	38–44 years	45–54 years	55–64 years	65–79 years
	AUC (IC_95%_)	AUC (IC_95%_)	AUC (IC_95%_)	AUC (IC_95%_)	AUC (IC_95%_)	AUC (IC_95%_)	AUC (IC_95%_)	AUC (IC_95%_)	AUC (IC_95%_)	AUC (IC_95%_)	AUC (IC_95%_)	AUC (IC_95%_)
Men												
Potassium (mEq/L)	0.54* (0.47–0.61)	0.54** (0.50–0.58)	0.53** (0.49–0.57)	0.52 (0.46–0.57)	0.68 (0.54–0.82)	0.57** (0.51–0.64)	0.55*** (0.49–0.60)	0.56* (0.52–0.61)	0.57* (0.51–0.64)	0.55*** (0.52–0.59)	0.54*** (0.51–0.57)	0.56* (0.52–0.59)
Creatinine (mg/dL)	0.53* (0.45–0.60)	0.58 (0.54–0.62)	0.58 (0.54–0.62)	0.52 (0.47–0.58)	0.71 (0.57–0.86)	0.57** (0.50–0.64)	0.57** (0.52–0.61)	0.57* (0.52–0.62)	0.57* (0.50–0.64)	0.58*** (0.55–0.62)	0.59*** (0.51–0.57)	0.57* (0.52–0.59)
HGS (*hand grip*)	0.64 (0.58–0.70)	0.61 (0.58–0.65)	0.60 (0.56–0.64)	0.54 (0.49–0.59)	0.76 (0.66–0.86)	0.70 (0.64–0.76)	0.66 (0.62–0.71)	0.63 (0.59–0.67)	0.67 (0.62–0.73)	0.65 (0.61–0.68)	0.64 (0.61–0.67)	0.62 (0.58–0.66)
Women												
Potassium (mEq/L)	0.48** (0.41–0.54)	0.56* (0.51–0.60)	0.53** (0.49–0.57)	0.59 (0.54–0.64)	0.51** (0.30–0.63)	0.54*** (0.47‐0.69)	0.60* (0.52–0.69)	0.53** (0.48–0.64)	0.48*** (0.48‐0.59)	0.55*** (0.52–0.54)	0.56*** (0.50–0.54)	0.58*** (0.52–0.67)
Creatinine (mg/dL)	0.53 (0.48–0.61)	0.61 (0.56–64)	0.55* (0.51–59)	0.67** (0.62–0.71)	0.59 (0.43–0.74)	0.59*** (0.53‐0.65)	0.60* (0.56–0.65)	0.62 (0.55–0.68)	0.56*** (0.50–0.62)	0.60*** (0.56–0.63)	0.58*** (0.55–0.61)	0.67*** (0.63–0.71)
HGS (*hand grip*)	0.60 (0.54–0.67)	0.63 (0.60–0.67)	0.62 (0.58–0.65)	0.57 (0.52–0.61)	0.76 (0.65–0.87)	0.74 (0.69–0.79)	0.68 (0.64–0.73)	0.68 (0.62–0.74)	0.64 (0.58–0.70)	0.68 (0.65–0.71)	0.65 (0.62–0.68)	0.62 (0.58–0.66)

*Note:* Test for differences between the area under the ROC curve of HGS and biomarkers of potassium and creatinine in urine in 24 h.

*** *p* < 0.001.

** *p* < 0.01.

* *p* < 0.05.

The cut‐off values obtained for HGS, although very close in the three analytical strategies used for estimation, were more discordant among women. Among men, it is possible to notice a greater disagreement of cut‐off points in the 55–64 age group. The cut‐off points that demonstrated greater accuracy and better performance in outcome discrimination were ≤ 42, ≤ 41, ≤ 38 and ≤ 36 kgf among males aged 38–44 years, 45–54 years, 55–64 years and 65–79 years, respectively. For women in the same age groups, HGS cut‐offs were ≤ 26, ≤ 23, ≤ 23 and ≤ 21 kgf, respectively. The cut‐off points that presented a better predictive power for sarcopenia and sarcopenic obesity with lower Brier scores when compared to other estimated points are shown in Table 4.

**TABLE 4:** Hand grip strength cut points estimated by different strategies, their respective values of sensitivity, specificity and Brier score for sarcopenia and sarcopenic obesity, according to the age range in men and women. ELSA‐Brasil, 2012–2014.

Strategies for analysis and validity measures	HGS Cutting points at ELSA‐Brasil							
	Men				Women			
	≤ 44 years	45–54 years	55–64 years	65–79 years	≤ 44 years	45–54 years	55–64 years	65–79 years
*J*								
Cut‐off	42.00	41.00	41.00	35.00	28.00	23.00	24.00	23.00
Sensitivity	0.61	0.61	0.47	0.62	0.43	0.69	0.53	0.48
Specificity	0.73	0.63	0.74	0.58	0.83	0.58	0.73	0.72
Brier score	0.34	0.36	0.44	0.39	0.46	0.28	0.38	0.44
*d* ^*2*^								
Cut‐off	42.00	41.00	38.00	35.00	26.00	24.00	23.0	21.00
Sensitivity	0.61	0.61	0.64	0.62	0.56	0.62	0.61	0.63
Specificity	0.73	0.64	0.57	0.58	0.68	0.65	0.63	0.55
Brier score	0.34	0.36	0.34	0.39	0.38	0.32	0.35	0.34
*e*								
Cut‐off	42.00	41.00	39.00	36.00	26.00	25.00	24.00	22.00
Sensitivity (%)	0.66	0.64	0.63	0.62	0.62	0.61	0.61	0.63
Specificity (%)	0.63	0.60	0.56	0.57	0.57	0.64	0.63	0.54
Brier score	0.34	0.36	0.38	0.39	0.38	0.37	0.38	0.38

*Note:* Youden's index (*J*) = maximal (sensitivity + specificity − 1). Less squared distance between the ROC curve (*d* ^2^) and the upper left corner of the graph of the area under the curve of higher sensitivity and specificity. Area under the curve of better balance between sensitivity and specificity (*e*).

Abbreviation: HGS = hand grip strength.

It was also observed that the risk of presenting sarcopenia and sarcopenic obesity is about 3 times greater in men with low HGS (HGS < 42.0) and less than 45 years and about two times higher in those with low HGS and in the age bands of 45 years or more. For women with low HGS, the odds of presenting sarcopenia and sarcopenic obesity ranged from 2.18 to 2.81 times between age groups. These results are shown in Table 5.

**TABLE 5:** Odds ratio (OR) and 95% confidence intervals (95% CI) of hand grip for the occurrence of sarcopenia and sarcopenic obesity, according to the age range in men and women. ELSA‐Brasil, 2012–2014.

HGS ‘optimal’ cut‐off points^a^		Sarcopenia and sarcopenic obesity		
	Total	*n*	%	OR (IC_95%_)
Men				
39–44 years (*n* = 593)				
HGS ≥ 42.0	367	34	9.26	1
HGS < 42.0	226	58	25.66	3.38 (2.12–5.63)
45–54 years (*n* = 2097)				
HGS ≥ 41.0	1270	118	9.29	1
HGS < 41.0	827	180	21.77	2.71 (2.11–3.49)
55–64 years (*n* = 1779)				
HGS ≥ 38.0	1160	204	17.59	1
HGS < 38.0	619	208	33.60	2.37 (1.89–2.97)
65–79 years (*n* = 962)				
HGS ≥ 35.0	567	172	30.34	1
HGS < 35.0	395	185	46.84	2.02 (1.54–2.64)
Women				
39–44 years (*n* = 649)				
HGS ≥ 26.0	386	38	9.84	1
HGS < 26.0	263	52	19.77	2.25 (1.43–3.54)
45–54 years (*n* = 2398)				
HGS ≥ 23.0	1713	149	8.99	1
HGS < 23.0	685	154	21.75	2.81 (2.20–3.59)
55–64 years (*n* = 2234)				
HGS ≥ 23.0	1402	155	11.06	1
HGS < 23.0	832	197	23.68	2.49 (1.98–3.14)
65–79 years (*n* = 1072)				
HGS ≥ 21.0	703	123	17.50	1
HGS < 21.0	369	117	31.71	2.18 (1.63–2.93)

Abbreviation: HGS = hand grip strength.

^a^ Cut‐off points that showed better performance and accuracy for estimated validity measures.

## 4 Discussion

The ELSA‐Brasil data revealed a good predictive capacity of the HGS test for discrimination of sarcopenia and sarcopenic obesity. Areas under the ROC curve with better performance in men and women were observed for isolated sarcopenic obesity (0.638 and 0.766), followed for the outcome analysed independently of the occurrence of obesity (0.624 and 0.681).

When comparing HGS performance with biomarkers of changes in muscle mass, potassium and urinary creatinine in 24 h, the data showed a predictive capacity of the HGS test in predicting the overall outcome of sarcopenia and sarcopenic obesity significantly higher in relation to the biomarkers, except between women in the age group above 65 years, in whom creatinine showed superior performance. Among those who presented sarcopenia in isolation, the differences between the tests were not expressive.

When comparing the performance of HGS with biomarkers of muscle mass changes, such as potassium and 24‐h urinary creatinine, the data demonstrated that the HGS test had a significantly higher predictive capacity for the overall outcome of sarcopenia and sarcopenic obesity, in comparison to the biomarkers. However, this was not the case for women above 65 years of age, where creatinine showed superior performance. Among those with isolated sarcopenia, the differences between the tests were not expressive.

The superior performance of HST in predicting sarcopenia when accompanied by obesity can be explained by its close relationship with muscular strength [1, 6, 28, 29]. Both sarcopenia and obesity are considered important markers of metabolic alterations [1, 7, 30]. This relationship is confirmed by data from a representative survey of Finnish adults, which showed a positive association between exposure to lifelong obesity exposure and low performance in the muscle strength test. This association points to the important role of early exposure to obesity in increasing the number of adults with sarcopenic obesity and metabolic disorders in future generations [29].

The strategies used to estimate HGS cut‐off points in the sarcopenia/sarcopenic obesity discrimination were concordant, especially among males in the 39–44 age group and 45–54 age group (42 and 41 kgf, respectively) and discordant in men in the age groups over 55 years and in women in all ranges, although this difference was small.

The HGS reference values for men and women across different age groups showed good sensitivity and acceptable specificity in discriminating sarcopenia and sarcopenic obesity [4, 8, 13, 28]. This underscores its recommendation as an initial screening tool for sarcopenia and sarcopenic obesity in adults [31, 32]. This could help prioritize care by identifying individuals requiring further evaluation.

The results of the present study showed HGS cut‐off points close to those verified in the literature in general health and physical performance prognoses [33, 34, 35]. They also corroborate the findings of Leong et al. in a longitudinal study of 139 691 adults aged 35 to 70 from 17 countries, who were representatively sampled in the Prospective Urban–Rural Epidemiology (PURE). The HGS values adjusted for age and height suggested by the authors for the general health prognosis were 30.2, 37.3 and 38.1 kgf in men and 24.3 and 27.9 and 26.6 kgf in women living in low‐, middle‐ and high‐income countries, respectively [10].

In Brazil, data from the survey ‘Nutrition, Physical Activity and Health’ (PNAFS) by Schlüssel et al., with a representative sample of 1122 men and 1928 adult women from the city of Niterói, Rio de Janeiro, Brazil, suggested normative values of 42.8 and 40.9 kgf for men and 25.3 and 24.0 kgf for women [36].

The current diagnostic consensus EWGSOP advocates the importance of assessing muscle strength in the diagnosis of sarcopenia and suggests cut‐offs of 37 and 21 kgf for men and women aged ≥ 65 years, respectively [5, 37]. The cut‐off point observed in women of the ELSA‐Brasil cohort in the same age group was similar to that suggested by consensus (21 kgf), a small variation was observed in men, whose reference value found was lower (36 kgf).

The variation between the cut‐off points estimated and those suggested in the literature, although small, can be explained by the differences in the anthropometric, ethnic, sociodemographic and lifestyle profile among the different population groups [3, 13, 38]. Such differences reiterate the importance of establishing specific reference values and with good predictive capacity for HGS measures to be used in population health prognoses. It is worth noting that the predictive capacity of HGS cut‐offs, evaluated through the Brier score, indicates a satisfactory overall performance of the prediction model.

The Brier score is a quadratic scoring rule that evaluates the overall performance of the model through the distance between the predicted probability and the probability of the occurrence of the outcome of interest. Thus, the smaller the distance, the lower the probability of classification error [27].

At present, there is a shortage of studies assessing the performance of the HGS compared to biomarkers as discriminators of sarcopenia in adult populations, which hinders result comparison. The data found resulted from studies in which different outcomes were used to assess the predictive capacity of this indicator.

A possible limitation of the study is the use of HGS as a proxy for total muscle strength and, as a parameter of hand function, may not accurately represent the strength performance achieved by other muscle groups, such as lower limbs and trunk. In addition, the assessment of muscle strength in all participants was performed with the Jamar dynamometer adjusted in a single position, which may represent an uncomfortable fit and influence test performance, especially among women who have a lower grip when compared to men. On the other hand, the adoption of adjustment in a single position has the advantage of standardizing the measurements, preserving the calibration of the equipment and facilitating the comparison of the data.

It is important to note that the cross‐sectional nature of the data implies a snapshot analysis of the variables at a single point in time. Furthermore, the results should be interpreted with caution, considering the lack of control over regular physical activity, especially resistance training, and the potential use of creatinine supplementation by participants in this study, which may have influenced the observed variability in the results. It is recommended that future research consider strategies to control these confounding variables in order to provide a more robust understanding of the predictive capacity of the HGS indicator. Although the age range of the reference population for SMMI estimation can be considered restricted, this is the first nationally representative study to provide normative values of normal muscle mass and sarcopenia based on data from a younger adult population as a reference, as well as establishes cut‐offs of muscular strength as discriminator of these conditions in adults of different age groups with potential of use in populations of other contexts and with characteristics similar to this one.

It is noteworthy that the good performance achieved by the HGS test when compared to the biochemical markers of creatinine and potassium in 24‐h urine samples reinforces its use in primary health care as a discriminator for the initial screening of muscle strength decline, sarcopenia and sarcopenic obesity. This potentially favours the anticipation of care strategies more appropriately tailored to the adult population. However, it is important to consider that the decline in HGS, as well as in SMMI, is not conclusive for the diagnosis of sarcopenia and sarcopenic obesity. Therefore, it should be used in conjunction with other indicators for a better definition of these conditions according to current scientific guidelines.

## 5 Conclusions

The results obtained in this study suggest that HGS, when compared to the performance of 24‐h urinary biomarkers of creatinine and potassium, demonstrated superior performance as a discriminator of sarcopenia and sarcopenic obesity in adults, especially in individuals under the age of 65. While the estimated cut‐off points showed good sensitivity and acceptable specificity for discriminating these conditions, it is important to highlight that HGS does not necessarily outperform other measures across all age groups, particularly in older age groups, where the incidence of sarcopenia tends to be higher.

In addition, the good performance achieved by the HGS test reinforces its use in primary health care as a tool to discriminator of sarcopenia and sarcopenic obesity, with the potential to anticipate care strategies for the population, especially in younger and middle‐aged adults.

## Ethics Statement

This study was conducted according to the guidelines laid down in the Declaration of Helsinki and all procedures involving research study participants were approved by the National Commission for Research Ethics and by the research ethics committees of all the six centers involved in these investigations: Hospital Universitário da Universidade de São Paulo (669/06); Hospital de Clínicas de Porto Alegre (06‐194); Fundação Oswaldo Cruz (343/06); Universidade Federal da Bahia (under the registration number 027‐06); Universidade Federal de Minas Gerais (186/06); and Universidade Federal do Espírito Santo (041/06).

## Consent

All participants signed the free and informed consent form, guaranteeing the secrecy and confidentiality of the data.

## Conflicts of Interest

The authors declare no conflicts of interest.

## Appendix A

### A.1

**FIGURE A1.** Areas under the ROC curve of HGS and biomarkers of muscle mass as discriminator of sarcopenia (isolated condition of SMMI < 1 standard deviation of the mean healthy young reference population), according to the age range in men and women. ELSA‐Brasil, 2012–2014.

**FIGURE A2.** Areas under the ROC curve of HGS and biomarkers of muscle mass as discriminator of sarcopenic obesity (isolated condition of SMMI < 1 standard deviation of the mean young reference population and BF > median specific sex in the total study population), according to the age range in men and women. ELSA‐Brasil, 2012–2014.

**FIGURE A3.** Areas under the ROC curve of HGS and biomarkers of muscle mass as discriminator Sarcopenia and sarcopenic obesity (combined outcome of sarcopenia + sarcopenic obesity), according to the age range in men and women. ELSA‐Brasil, 2012–2014.

## References

[1] C.‐L. Cheung , U.‐S. D. T. Nguyen , E. Au , K. C. B. Tan , and A. W. C. Kung , “Association of Handgrip Strength With Chronic Diseases and Multimorbidity: A Cross‐Sectional Study,” Age (Dordrecht, Netherlands) 35, no. 3 (2013): 929–941. 22314403 10.1007/s11357-012-9385-y PMC3636411

[2] A. A. Sayer and T. B. Kirkwood , “Grip Strength and Mortality: A Biomarker of Ageing?,” Lancet 386, no. 9990 (2015): 226–227. 25982159 10.1016/S0140-6736(14)62349-7

[3] C. A. Jackson , A. Dobson , L. Tooth , and G. D. Mishra , “Body Mass Index and Socioeconomic Position Are Associated With 9‐Year Trajectories of Multimorbidity: A Population‐Based Study,” Preventive Medicine 81 (2015): 92–98. 26311587 10.1016/j.ypmed.2015.08.013

[4] P. M. Cawthon , “Special Section: Sarcopenia Assessment of Lean Mass and Physical Performance in Sarcopenia,” Journal of Clinical Densitometry 18, no. 4 (2015): 467–471. 26071168 10.1016/j.jocd.2015.05.063

[5] A. J. Cruz‐Jentoft , G. Bahat , J. Bauer , et al., “Sarcopenia: Revised European Consensus on Definition and Diagnosis,” Age and Ageing 48, no. 1 (2019): 16–31. 30312372 10.1093/ageing/afy169 PMC6322506

[6] K. Sakuma and A. Yamaguchi , “Sarcopenic Obesity and Endocrinal Adaptation With Age,” International Journal of Endocrinology 2013 (2013): 1–12. 10.1155/2013/204164 PMC3639625 23690769

[7] J. A. Cauley , “An Overview of Sarcopenic Obesity,” Journal of Clinical Densitometry 18, no. 4 (2015): 499–505. 26141163 10.1016/j.jocd.2015.04.013

[8] M. Pahor and M. Cesari , “Sarcopenia: Clinical Evaluation, Biological Markers and Other Evaluation Tools,” Journal of Nutrition, Health & Aging 13, no. 8 (2009): 724–728. 10.1007/s12603-009-0204-9 PMC4312657 19657557

[9] S. B. Heymsfield , M. C. Gonzalez , J. Lu , G. Jia , and J. Zheng , “Skeletal Muscle Mass and Quality: Evolution of Modern Measurement Concepts in the Context of Sarcopenia,” Proceedings of the Nutrition Society 74, no. 4 (2015): 355–366. 25851205 10.1017/S0029665115000129

[10] M. Tosato , E. Marzetti , M. Cesari , et al., “Measurement of Muscle Mass in Sarcopenia: From Imaging to Biochemical Markers,” Aging Clinical and Experimental Research 29 (2017): 19–27. 28176249 10.1007/s40520-016-0717-0

[11] D. P. Leong , K. K. Teo , S. Rangarajan , et al., “Prognostic Value of Grip Strength: Findings From the Prospective Urban Rural Epidemiology (PURE) Study,” Lancet 386, no. 9990 (2015): 266–273. 25982160 10.1016/S0140-6736(14)62000-6

[12] S. Stenholm , N. K. Mehta , I. T. Elo , M. Heliövaara , S. Koskinen , and A. Aromaa , “Obesity and Muscle Strength as Long‐Term Determinants of All‐Cause Mortality–A 33‐Year Follow‐Up of the Mini‐Finland Health Examination Survey,” International Journal of Obesity 38, no. 8 (2014): 1126–1132. 24232499 10.1038/ijo.2013.214 PMC4022712

[13] D. E. Alley , M. D. Shardell , K. W. Peters , et al., “Grip Strength Cutpoints for the Identification of Clinically Relevant Weakness,” Journals of Gerontology. Series A, Biological Sciences and Medical Sciences 69, no. 5 (2014): 559–566. 24737558 10.1093/gerona/glu011 PMC3991145

[14] S. Barbat‐Artigas , S. Plouffe , C. H. Pion , and M. Aubertin‐Leheudre , “Toward a Sex‐Specific Relationship Between Muscle Strength and Appendicular Lean Body Mass Index?,” Journal of Cachexia, Sarcopenia and Muscle 4, no. 2 (2013): 137–144. 23389764 10.1007/s13539-012-0100-8 PMC3684704

[15] E. M. L. Aquino , S. M. Barreto , I. M. Bensenor , et al., “Brazilian Longitudinal Study of Adult Health (ELSA‐Brasil): Objectives and Design,” American Journal of Epidemiology 175, no. 4 (2012): 315–324. 22234482 10.1093/aje/kwr294

[16] D. Chor , M. G. Alves , L. Giatti , et al., “Questionnaire Development in ELSA‐Brasil: Challenges of a Multidimensional Instrument,” Revista de Saúde Pública 47, no. Suppl 2 (2013): 27–36. 24346718 10.1590/s0034-8910.2013047003835

[17] T. G. Lohman , A. F. Roche , and R. Martorell , Anthropometric Standardization Reference Manual (Human Kinetics Books, 1988): 55–68.

[18] A. D. Karelis , G. Chamberland , M. Aubertin‐Leheudre , and C. Duval , “Validation of a Portable Bioelectrical Impedance Analyzer for the Assessment of Body Composition,” Applied Physiology, Nutrition, and Metabolism 38, no. 1 (2013): 27–32. 10.1139/apnm-2012-0129 23368825

[19] H. C. Roberts , H. J. Denison , H. J. Martin , et al., “A Review of the Measurement of Grip Strength in Clinical and Epidemiological Studies: Towards a Standardised Approach,” Age and Ageing 40 (2011): 423–429. 21624928 10.1093/ageing/afr051

[20] L. G. Fedeli , P. G. Vidigal , C. M. Leite , et al., “Logistics of Collection and Transportation of Biological Samples and the Organization of the Central Laboratory in the ELSA‐Brasil,” Revista de Saúde Pública 47, no. Suppl 2 (2013): 63–71. 24346722 10.1590/s0034-8910.2013047003807

[21] I. Janssen , D. S. Shepard , P. T. Katzmarzyk , and R. Roubenoff , “The Healthcare Costs of Sarcopenia in the United States,” Journal of the American Geriatrics Society 52, no. 1 (2004): 80–85. 14687319 10.1111/j.1532-5415.2004.52014.x

[22] I. Janssen , S. B. Heymsfield , Z. M. Wang , and R. Ross , “Skeletal Muscle Mass and Distribution in 468 men and Women Aged 18‐88 Yr,” Journal of Applied Physiology 89, no. 1 (2000): 81–88. 10904038 10.1152/jappl.2000.89.1.81

[23] World Health Organization . “WHO Study Group on Assessment of Fracture Risk and Its Application to Screening for Postmenopausal Psteoporosis: Report of a WHO Study Group,” (1994). 7941614

[24] R. N. Baumgartner , K. M. Koehler , D. Gallagher , et al., “Epidemiology of Sarcopenia Among the Elderly in New Mexico,” American Journal of Epidemiology 147, no. 8 (1998): 755–763. 9554417 10.1093/oxfordjournals.aje.a009520

[25] J. S. Kim , J. M. Wilson , and S. R. Lee , “Dietary Implications on Mechanisms of Sarcopenia: Roles of Protein, Amino Acids and Antioxidants,” Journal of Nutritional Biochemistry 21, no. 1 (2010): 1–13. 19800212 10.1016/j.jnutbio.2009.06.014

[26] N. J. Perkins and E. F. Schisterman , “The Inconsistency of “Optimal” Cut‐Points Using Two ROC Based Criteria,” American Journal of Epidemiology 163, no. 7 (2006): 670–675. 16410346 10.1093/aje/kwj063 PMC1444894

[27] E. W. Steyerberg , A. J. Vickers , N. R. Cook , et al., “Assessing the Performance of Prediction Models: A Framework for Some Traditional and Novel Measures,” Epidemiology 21, no. 8 (2010): 128–138. 20010215 10.1097/EDE.0b013e3181c30fb2 PMC3575184

[28] G. Biolo , T. Cederholm , and M. Muscaritoli , “Muscle Contractile and Metabolic Dysfunction Is a Common Feature of Sarcopenia of Aging and Chronic Diseases: From Sarcopenic Obesity to cachexia,” Clinical Nutrition 33, no. 5 (2014): 737–748. 24785098 10.1016/j.clnu.2014.03.007

[29] S. Stenholm , J. Sallinen , A. Koster , et al., “Association Between Obesity History and Hand Grip Strength in Older Adults–Exploring the Roles of Inflammation and Insulin Resistance as Mediating Factors,” Journals of Gerontology. Series A, Biological Sciences and Medical Sciences 66A, no. 3 (2011): 341–348. 10.1093/gerona/glq226 PMC3041473 21310808

[30] A. A. Sayer , H. E. Syddall , E. M. Dennison , et al., “Grip Strength and the Metabolic Syndrome: Findings From the Hertfordshire Cohort Study,” QJM 100, no. 11 (2007): 707–713. 17951315 10.1093/qjmed/hcm095 PMC2292249

[31] G. Tripepi , K. J. Jager , F. W. Dekker , and C. Zoccali , “Diagnostic Methods 2: Receiver Operating Characteristic (ROC) Curves,” Kidney International 76, no. 3 (2009): 252–256. 19455194 10.1038/ki.2009.171

[32] K. J. Van Stralen , V. S. Stel , J. B. Reitsma , F. W. Dekker , C. Zoccali , and K. J. Jager , “Diagnostic Methods I: Sensitivity, Specificity, and Other Measures of Accuracy,” Kidney International 75, no. 12 (2009): 1257–1263. 19340091 10.1038/ki.2009.92

[33] R. M. Dodds , H. E. Syddall , R. Cooper , D. Kuh , C. Cooper , and A. A. Sayer , “Global Variation in Grip Strength: A Systematic Review and Meta‐Analysis of Normative Data,” Age and Ageing 45, no. 2 (2016): 209–216. 26790455 10.1093/ageing/afv192 PMC4776623

[34] R. W. Bohannon , A. Peolsson , N. Massy‐Westropp , J. Desrosiers , and J. Bear‐Lehman , “Reference Values for Adult Grip Strength Measured With a Jamar Dynamometer: A Descriptive meta‐Analysis,” Physiotherapy 92, no. 1 (2006): 11–15.

[35] R. W. Bohannon and S. Magasi , “Identification of Dynapenia in Older Adults Through the Use of Grip Strength t‐Scores,” Muscle & Nerve 51, no. 1 (2015): 102–105. 24729356 10.1002/mus.24264 PMC4194263

[36] M. M. Schlüssel , L. A. dos Anjos , M. T. L. de Vasconcellos , and G. Kac , “Reference Values of Handgrip Dynamometry of Healthy Adults: A Population‐Based Study,” Clinical Nutrition 27 (2008): 601–607. 18547686 10.1016/j.clnu.2008.04.004

[37] R. M. Dodds , H. E. Syddall , R. Cooper , et al., “Grip Strength Across the Life Course: Normative Data From Twelve British Studies,” PLoS ONE 9, no. 12 (2014): e113637. 25474696 10.1371/journal.pone.0113637 PMC4256164

[38] S. A. Haas , P. M. Krueger , and L. Rohlfsen , “Race/Ethnic and Nativity Disparities in Later Life Physical Performance: The Role of Health and Socioeconomic Status Over the Life Course,” Journals of Gerontology. Series B, Psychological Sciences and Social Sciences 67, no. 2 (2012): 238–248. 22391749 10.1093/geronb/gbr155 PMC3410696

